# The evolution of queen control over worker reproduction in the social Hymenoptera

**DOI:** 10.1002/ece3.3324

**Published:** 2017-09-10

**Authors:** Jason Olejarz, Carl Veller, Martin A. Nowak

**Affiliations:** ^1^ Program for Evolutionary Dynamics Harvard University Cambridge MA USA; ^2^ Department of Organismic and Evolutionary Biology Harvard University Cambridge MA USA; ^3^ Department of Mathematics Harvard University Cambridge MA USA

**Keywords:** chemical communication, evolutionary dynamics, natural selection, pheromones, reproductive division of labor, social insects

## Abstract

A trademark of eusocial insect species is reproductive division of labor, in which workers forego their own reproduction while the queen produces almost all offspring. The presence of the queen is key for maintaining social harmony, but the specific role of the queen in the evolution of eusociality remains unclear. A long‐discussed scenario is that a queen either behaviorally or chemically sterilizes her workers. However, the demographic and ecological conditions that enable such manipulation are still debated. We study a simple model of evolutionary dynamics based on haplodiploid genetics. Our model is set in the commonly observed case where workers have lost the ability to lay female (diploid) eggs by mating, but retain the ability to lay male (haploid) eggs. We consider a mutation that acts in a queen, causing her to control the reproductive behavior of her workers. Our mathematical analysis yields precise conditions for the evolutionary emergence and stability of queen‐induced worker sterility. These conditions do not depend on the queen's mating frequency. We find that queen control is always established if it increases colony reproductive efficiency, but can evolve even if it decreases colony efficiency. We further derive the conditions under which queen control is evolutionarily stable against invasion by mutant workers who have recovered the ability to lay male eggs.

## 1 INTRODUCTION

Many species of ants, bees, and wasps form highly complex eusocial societies characterized by dominance hierarchies and reproductive division of labor (Gadagkar, 2001; Hӧlldobler & Wilson, 1990; Hunt, 2007; Michener, 1974; Wilson, 1971). In most cases, both the queen and the workers are capable of laying male eggs parthenogenetically, but the workers often forego their own reproduction, allowing the queen to produce the majority of drones (Bourke, 1988; Fletcher & Ross, 1985; Heinze, 2004; Ratnieks, Foster, & Wenseleers, 2006; Wilson, 1971).

There are several ways in which this behavior could arise. One possibility is that a “policing” mutation acts in a worker, causing that worker to destroy male eggs produced by other workers (Olejarz, Allen, Veller, Gadagkar, & Nowak, 2016; Ratnieks, 1988). Alternatively, a “nonreproduction” mutation could act in a worker, causing that worker to forego its own reproduction (Doebeli & Abouheif, 2015; Olejarz, Allen, Veller, & Nowak, 2015). Such mutations can spread and eventually fix in the population if the resulting gains in colony reproductive efficiency are sufficiently large (Olejarz et al., 2015, 2016; Ratnieks, 1988). In yet another scenario, a mutation could act in a queen, causing her to suppress her workers' reproduction (Bourke, 1988; Charlesworth, 1978; Craig, 1979; Hӧlldobler & Wilson, 1990; Vienne, Errard, & Lenoir, 1998). Here, we study the population genetics of this possibility.

There are several mechanisms by which a queen can manipulate her workers' reproductive output (reviewed in Ronai, Vergoz, and Oldroyd (2016b)). In small colonies, the queen (or, more generally, the dominant individual) can directly control worker reproduction by eating worker‐laid eggs or by aggressing workers who attempt to lay eggs (Bourke & Franks, 1995; Dapporto, Bruschini, Cervo, Petrocelli, & Turillazzi, 2010; Heinze, Hӧlldobler, & Peeters, 1994; Koedam, Brone, & van Tienen, 1997; Michener, 1974; Oster & Wilson, 1978; Smith, Hӧlldobler, & Liebig, 2011; Wilson, 1971). Indirect chemical suppression of worker reproduction is also possible through queen pheromones (Keller & Nonacs, 1993; Konrad, Pamminger, & Foitzik, 2012; Leonhardt, Menzel, Nehring, & Schmitt, 2016; Nunes et al., 2014; Oi, Van Oystaeyen, et al., 2015; Richard & Hunt, 2013), which are especially important in the large colonies of highly eusocial species, where direct queen policing is infeasible (Fletcher & Ross, 1985; Gadagkar, 1997; Katzav‐Gozansky, 2006; Le Conte & Hefetz, 2008).

Pheromonal suppression by queens or dominant individuals has long been recognized in the eusocial Hymenoptera (Butler & Simpson, 1958; Keller & Nonacs, 1993; Kocher & Grozinger, 2011). For example, queen tergal gland secretions (Wossler & Crewe, 1999) and queen mandibular pheromone (Hoover, Keeling, Winston, & Slessor, 2003; Ronai, Oldroyd, & Vergoz, 2016c; Ronai, Oldroyd, et al., 2016a) have both been shown to limit ovarian development in honeybee workers (genus *Apis*), while in the carpenter ant *Camponotus floridanus*, worker‐laid eggs experimentally marked with the queen‐derived surface hydrocarbons were significantly less likely to be destroyed by other workers (Endler et al., 2004). Pheromonal suppression of worker reproduction has also been documented in primitively eusocial species, including the polistine wasps *Polistes dominula* (Sledge, Boscaro, & Turillazzi, 2001) and *Ropalidia marginata* (Bhadra et al., 2010; Mitra, 2014; Saha et al., 2012), the euglossine bee *Euglossa melanotricha* (Andrade‐Silva & Nascimento, 2015), and several species in *Bombus* (Ayasse & Jarau, 2014; Holman, 2014).

Despite the ubiquity of the phenomenon, a comprehensive theoretical understanding of the evolution of queen suppression of worker reproduction is lacking. What are the precise conditions under which queen control evolves? What demographic and ecological characteristics of insect populations result in the evolutionary emergence of queen control? To address these questions, we formulate a model of population dynamics that is based on haplodiploid genetics (Nowak, Tarnita, & Wilson, 2010; Olejarz et al., 2015, 2016). Our model takes as context a species in which workers can lay unfertilized (male) eggs, but do not mate, and therefore cannot lay fertilized (female) eggs. (This situation is especially common in the higher eusocial Hymenoptera (Bourke, 1988; Fletcher & Ross, 1985), where workers in many species have retained functional ovaries, but have lost the ability to mate because of physiological factors like degradation of the spermatheca or diminution of the bursa copulatrix. It is also a common situation in primitively eusocial bees and wasps (Fletcher & Ross, 1985), where workers often retain the physiological capability of mating, but nonetheless do not mate because of an absence of males at the relevant stage of the colony life cycle, or for behavioral reasons.) In this model, we study the population genetics of alleles, dominant or recessive, that act in queens to reduce worker reproduction. Within our setup, we derive exact conditions for invasion and stability of these alleles, for any number of matings of the queen, and interpret these conditions in terms of the colony efficiency effects of suppressing worker reproduction.

A related, long‐standing debate in the literature concerns the nature of queen chemical suppression of worker reproduction in terms of workers' “evolutionary interests” (Heinze & d'Ettorre, 2009; Keller & Nonacs, 1993; Le Conte & Hefetz, 2008). Should queen chemical suppression be interpreted as coercive control of workers (against their evolutionary interests), or are these chemicals best thought of as honest signals of queen presence or fertility (so that their induction of nonreproduction in workers can in fact be in the workers' evolutionary interests)? Empirical studies have provided support for both interpretations (Brunner, Kroiss, Trindl, & Heinze, 2011; Heinze & d'Ettorre, 2009; Holman, 2010; Katzav‐Gozansky, 2006; Keller & Nonacs, 1993; Kocher & Grozinger, 2011; Kocher, Richard, Tarpy, & Grozinger, 2009; Le Conte & Hefetz, 2008; Maisonnasse et al., 2010; Peso, Elgar, & Barron, 2015; Strauss et al., 2008; van Zweden, 2010).

Our setup, based on population genetics, offers a simple framework for classifying queen suppressor chemicals as either coercion or honest signals. Suppose a queen suppressor mutation has fixed, so that all queens produce chemicals that suppress workers' reproduction. Now suppose that a “resistance” mutation arises that renders workers in whom it is expressed immune to queen suppressor chemicals, so that these workers again lay male eggs. If this “resistance” mutation invades, then resistance is seen to be in the workers' evolutionary interests, and the initial queen suppression should be interpreted as coercive. If not, then we interpret the queen suppressor chemical to be an honest signal (González‐Forero & Gavrilets, 2013). Invadability of the population by this rare “resistance” allele is equivalent to evolutionary instability of a nonreproduction allele acting in workers, the formal population genetical conditions for which are given in Olejarz et al. (2015). We use these conditions to distinguish the demographic and ecological parameter regimes in which queen suppression should be thought of as coercion or as honest signaling. We also explore the similarly relevant possibility of partial queen control—where the queen prevents some, but not all, of workers' reproduction—subsequently inducing complete worker sterility (Bourke, 1988; Ratnieks et al., 2006).

## 2 MODEL

Haplodiploidy, the genetic system in which males are haploid while females are diploid, is neither a necessary nor a sufficient precondition for the emergence of eusociality. Some eusocial species are diploid, such as termites (Alexander, 1974), some mole‐rats (Jarvis & Bennett, 1993), and some shrimp (Duffy, Morrison, & Ruben, 2000), while the eusocial Hymenoptera (ants, bees, and wasps) are haplodiploid. Our model is set in a haplodiploid species. Fertilized eggs (diploid) become females, and unfertilized eggs (haploid) become males.

**Figure 1:**
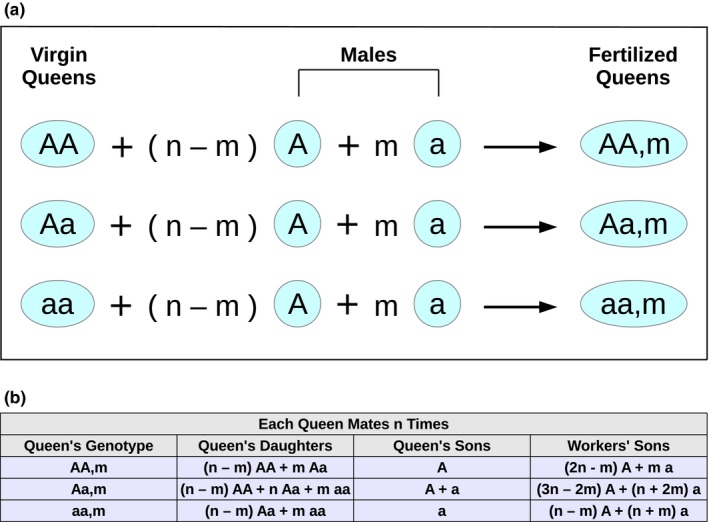
The mating events are shown in (a). The reproduction events are shown in (b)

To start, consider a large population of colonies, where each colony is headed by either a queen or a dominant individual, and each colony contains many females and males. Most of the females stay at the natal nest as workers, but a small number of females act as gynes, leaving the natal nest to mate with one or more males from other colonies in the population. A single gyne mates with *n* distinct drones that are chosen randomly among all drones in the population. She then founds a new colony and assumes the dominant position within her colony (Figure 1a). She fertilizes haploid eggs with the sperm from each of the *n* males that she mated with to produce diploid female eggs. When these female eggs hatch, many of the resulting individuals become workers in the colony, while some become gynes. In addition, the queen or dominant individual produces unfertilized haploid male eggs. Workers can also produce haploid male eggs; this leads to reproductive conflict over male production within a colony (Figure 1b).

We consider the evolutionary dynamics of two alleles—a wild‐type allele, *A*, and a mutant allele, *a* that, when expressed in queens, causes them to suppress their workers' male production. We use the following notation for individuals of various genotypes. There are two types of drones: *A* and *a*. There are three types of gynes: *AA*,* Aa*, and *aa*. A queen's type (or, equivalently, that of a colony, as each colony is headed by a single queen) is denoted *AA*,* m*;* Aa*,* m*; or *aa*,* m*, depending on whether the queen's own genotype is *AA*,* Aa*, or *aa*, respectively, and the number, *m*, of mutant (type *a*) drones she mated with, requiring 0 ≤ *m *≤ *n*. We use the notation *X*_*AA, m*_, *X*_*Aa, m*_, and *X*_*aa, m*_ to denote the frequencies of the colony types in the population, requiring $\sum_{m=0}^{n}\left(X_{AA,m}+X_{Aa,m}+X_{aa,m}\right)=1$ at all times.

If the mutant allele, *a*, is dominant, then type *AA*,* m* queens are wild‐type, while type *Aa*,* m* and type *aa*,* m* queens have the mutant phenotype. If the mutant allele, *a*, is recessive, then type *AA*,* m* and type *Aa*,* m* queens are wild‐type, while type *aa*,* m* queens have the mutant phenotype.

In colonies headed by wild‐type queens, a fraction 0 ≤ *p *≤ 1 of males are produced by the queen (so that a fraction 1 − *p* of males are produced by the workers), and new gynes and drones are produced at rate *r *≥ 0. In colonies headed by queens with the mutant phenotype, a fraction 0 ≤ *p*′ ≤ 1 of males are produced by the queen (so that a fraction 1 − *p*′ of males are produced by the workers), and new gynes and drones are produced at rate *r*′ ≥ 0. Thus, colonies headed by queens with the mutant phenotype have different values of the fraction of queen‐produced males and colony efficiency—*p*′ and *r*′, respectively—compared with colonies headed by wild‐type queens.

Derivations are provided in the Supporting Information. We shall show that, under the assumptions we have made, these empirical quantities, *p*,* r*,* p*′, and *r*′, are sufficient to predict whether the queen‐control allele, *a*, can invade, and whether it is resistant to invasion when fixed. In principle, these colony‐level quantities are directly measurable: How many reproductive males do the two colony types produce, and what proportion of these are produced by workers in each case? Clearly, *p*,* r*,* p*′, and *r*′ result from the interplay between many demographic and ecological factors, but these need not be known to predict the fate of a queen‐control allele. It is instructive to consider the relative values of these parameters in the context of a queen that influences her workers' reproduction. We expect that *p*′ > *p*; that is, the effect of the queen's manipulation is to increase the fraction of male eggs that come from her. *r*′ may be greater than or less than *r*. If *r*′ > *r*, then the queen's manipulation effects an increase in colony efficiency, while if *r*′ < *r*, then the queen's manipulation effects a decrease in colony efficiency.

Of course, queen inhibition of parthenogenetic worker reproduction is just one of many eusocial traits. It is unlikely that queen manipulation of worker reproduction only took hold after workers lost the ability to mate and reproduce sexually. For the sake of mathematical modeling, we may accordingly loosen our interpretation of the “queen” to mean any individual that mates with *n* males and produces many male and female offspring to form a new colony. In other words, our model does not necessitate that a mated, sexually reproductive female be morphologically distinct from any other female in the population. The modeling herein can therefore be applied toward understanding the development of queen suppression of asexual worker reproduction in many primitively eusocial species as well as advanced eusocial species.

Furthermore, although our analysis assumes that all colonies have the same sex ratio, the sex ratio itself does not factor into our analysis. In other words, regardless of the particular value of the sex ratio that one assumes, the sex ratio affects only the overall timescale; it does not alter the evolutionary trajectories as prescribed by our model.

We briefly note the following limitations of our analysis. If there are overlapping matrilines within a colony, that is, if colonies are polygynous, or if the notion of a colony headed by a single dominant individual is not well defined, then our model is not directly applicable. Moreover, the problem of nest formation would require different modeling considerations and is therefore not treated here. We additionally note that the problem of the evolution of queen control in diploid species lies outside the scope of this work.

## 3 RESULTS

### 3.1 The evolution of queen control

In simplest mathematical terms, the key question is as follows: What values of the parameters *p*,* r*,* p′*, and *r*′ support the evolution of queen suppression of workers' reproduction? We derive the following main results.

The *a* allele, which causes the queen to suppress her workers' reproduction, invades a population of noncontrolling queens if the following condition holds:

(1) $$\frac{r^{\prime }}{r}>1-\frac{p^{\prime }-p}{2+p+p^{\prime }},$$

**Figure 2:**
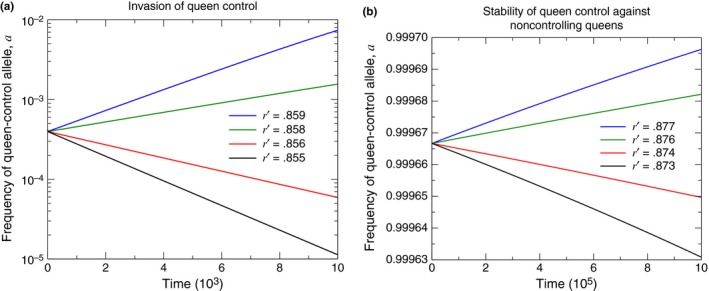
Numerical simulations demonstrate the condition for evolutionary invasion of queen control ((a), Condition (1)) and the condition for queen control to be evolutionarily stable, when fixed, against invasion by noncontrolling queens ((b), Condition (2)). For these plots, we consider a dominant queen‐control allele with singly mated queens (*n* = 1), and we set *p* = .5, *p*′ = 1, and *r* = 1. (The initial conditions are (a) *X*_*AA*, 0_ = 1 − 10^−3^ and *X*_*AA*, 1_ = 10^−3^ for each of the four curves, and (b) *X*_*aa*, 1_ = 1 − 10^−3^ and *X*_*aa*, 0_ = 10^−3^ for each of the four curves.)

Condition (1) applies regardless of whether the queen‐control allele, *a*, is dominant or recessive. The evolutionary dynamics demonstrating Condition (1) for single mating and for a dominant queen‐control allele are shown in Figure 2(a).

Furthermore, the queen‐control allele, *a*, when fixed in the population, is stable against invasion by the noncontrolling *A* allele if the following condition holds:

(2) $$\frac{r^{\prime }}{r}>1-\frac{p^{\prime }-p}{2(1+p^{\prime })},$$

Condition (2) also applies regardless of whether the queen‐control allele, *a*, is dominant or recessive. The evolutionary dynamics demonstrating Condition (2) for single mating and for a dominant queen‐control allele are shown in Figure 2(b).

**Figure 3:**
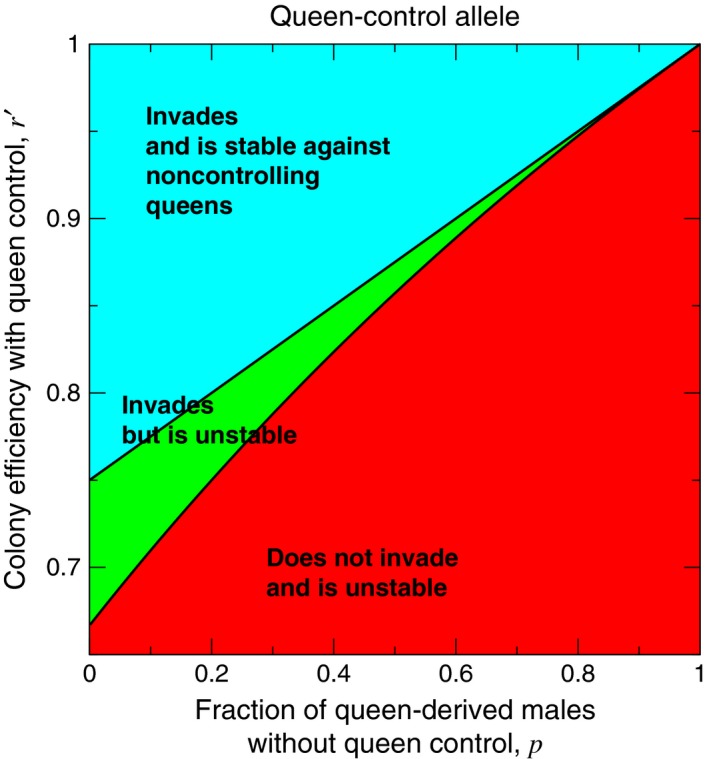
A plot of *r*′ versus *p* shows the three possibilities for the dynamical behavior of the queen‐control allele around the two pure equilibria. For this plot, we set *r* = 1 and *p*′ = 1

**Figure 4:**
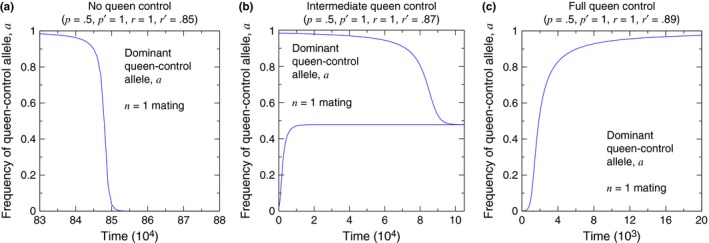
Simulations of the evolutionary dynamics show the dynamical behavior of the queen‐control allele. (a) corresponds to the lower, red region in Figure 3. (b) corresponds to the middle, green region in Figure 3. (c) corresponds to the upper, blue region in Figure 3. (The initial conditions are (a) *X*_*aa*, 1_ = 1 − 10^−3^ and *X*_*aa*, 0_ = 10^−3^, (b, lower curve) *X*_*AA*, 0_ = 1 − 0.05 and *X*_*AA*, 1_ = 0.05, (b, upper curve) *X*_*aa*, 1_ = 1 − 0.05 and *X*_*aa*, 0_ = 0.05, and (c) *X*_*AA*, 0_ = 1 − 10^−3^ and *X*_*AA*, 1_ = 10^−3^.)

If *p*′ > *p*, then Condition (1) is always easier to satisfy than Condition (2). Therefore, three scenarios regarding the two pure equilibria are possible: The first possibility is that queen control is unable to invade a wild‐type population and is unstable, when fixed, against invasion by noncontrol. The second possibility is that queen control is able to invade a wild‐type population but is unstable, when fixed, against invasion by noncontrol. The third possibility is that queen control is able to invade a wild‐type population and is stable, when fixed, against invasion by noncontrol. In the case where queen control can invade a wild‐type population but is unstable when fixed, Brouwer's fixed‐point theorem guarantees the existence of at least one mixed equilibrium at which controlling and noncontrolling queens coexist. Regions of the parameter space are shown in Figure 3, and evolutionary dynamics illustrating the three scenarios are shown in Figure 4.

Two points regarding the dynamics of the queen‐control allele deserve emphasis. First, the conditions for evolutionary invasion and stability of queen control do not depend on the queen's mating number, *n*. To develop intuition, consider the introduction of an initially rare dominant allele for queen control. When the allele is rare, for *n* matings, and after sufficient time has elapsed, the ratio of the frequency of *AA*, 1 colonies to the frequency of *Aa*, 0 colonies includes a factor of *n*. A fraction (*n *− 1)/*n* of offspring of *AA*, 1 colonies arise from selecting sperm from wild‐type males and are 100% wild‐type, as though they had originated from *AA*, 0 colonies. However, the remaining fraction 1/*n* of offspring of *AA*, 1 colonies are produced in the same relative mutant/wild‐type proportions as if they had originated from *AA*,* n* colonies. Notice that the factor of *n* from the matings cancels with the probability of 1/*n* of selecting sperm from the mutant male. Therefore, we have a simple interpretation: For considering invasion of queen control, and at the leading‐order frequency of the mutant allele, the system effectively consists of *AA*,* n* colonies and *Aa*, 0 colonies at relative amounts that do not depend on *n*. But *AA*,* n* colonies produce mutant and wild‐type offspring in relative proportions that do not depend on *n*, and *Aa*, 0 colonies produce mutant and wild‐type offspring in relative proportions that do not depend on *n*. Thus, *n* does not enter into Condition (1). (However, the number of matings, *n*, does affect the evolutionary dynamics at intermediate frequencies of the queen‐control allele.)

Second, queen control can evolve even if it results in efficiency losses. This can be seen from Conditions (1) and (2), where, in both cases, the right‐hand side is less than 1 because *p*′ > *p*. A simple relatedness‐based argument already suggests this (Bourke, 1988), as the queen has relatedness 1/2 to her sons and relatedness 1/4 to her grandsons. More precisely, consider the transmission of the mutant allele for the cases of dominant and recessive queen‐control alleles:

If the queen‐control allele is dominant, then type *Aa*, 0 (and type *aa*, 0) colonies have the mutant phenotype. In the dominant case, type *Aa*, 0 colonies produce type *AA* and type *Aa* workers in equal proportion, so workers in type *Aa*, 0 colonies produce 3 type *A* males for every type *a* male. But the queen produces type *A* and type *a* males in equal proportion. Therefore, notice that if a wild‐type queen produces only some males (0 ≤ *p* < 1) and a mutant queen produces a greater fraction of males (*p* < *p*′ ≤ 1), and if all else is the same, then colonies headed by mutant *Aa*, 0 queens will produce a larger relative amount of the mutant allele in their offspring than wild‐type colonies. So it can be the case that mutant *Aa*, 0 colonies have a slightly lower overall reproductive efficiency than wild‐type colonies while still increasing the relative amount of the mutant allele in the population.

If the queen‐control allele is recessive, then type *aa*, 0 colonies have the mutant phenotype. In the recessive case, type *aa*, 0 colonies produce only type *Aa* workers, so workers in type *aa*, 0 colonies produce type *A* and type *a* males in equal proportion. But the queen produces only type *a* males. Therefore, just as for the case of a dominant queen‐control allele, notice that if a wild‐type queen produces only some males (0 ≤ *p* < 1) and a mutant queen produces a greater fraction of males (*p* < *p*′ ≤ 1), and if all else is the same, then colonies headed by mutant *aa*, 0 queens will produce a larger relative amount of the mutant allele in their offspring than wild‐type colonies. So, again, it can be that mutant *aa*, 0 colonies have a lower overall reproductive efficiency than wild‐type colonies while still increasing the relative amount of the mutant allele in the population.

Thus, the allele for queen control can act as a selfish genetic element (Burt & Trivers, 2006), enabling queen‐induced worker sterility to develop in a population even if it diminishes colony reproductive efficiency. (This is consistent with earlier work of Craig (1979), although that work considered parental manipulation in a different context, in which workers either reproduce sexually or become helpers of their queen. Avila and Fromhage (2015) also found that synergistic efficiency gains from helping are not necessary for evolution of sterile workers, but they too consider a different setup, whereby nest‐site limitation and dispersal mortality act as ecological constraints that promote the evolution of eusociality.)

### 3.2 Worker resistance or acquiescence: is queen control coercive or an honest signal?

We have shown that queens are easily selected to increase their production of male offspring and suppress workers' production of male offspring. In this case, workers might also be selected to evade manipulation by queens, setting up an evolutionary arms race. When does queen control evolve and persist in the population?

Consider the following scenario. Initially, there is a homogeneous population of colonies. The allele *A* is fixed at locus A, and the allele *B* is fixed at locus B. In each colony, the fraction of queen‐derived males within the colony is *p*, and the overall reproductive efficiency of the colony is *r*. Suppose that a mutation at the A locus, *a*, acts in a queen, causing her to completely suppress her workers' production of drones. In colonies headed by controlling queens, all males originate from the controlling queen (*p*′ = 1), and the overall reproductive efficiency of the colony is *r*′. According to Conditions (1) and (2), if *r*′/*r* is sufficiently large (>[3 + *p*]/4), then the queen‐control allele will increase in frequency and fix in the population. Once the queen‐control allele has fixed, each colony's male eggs originate only from the queen (*p*′ = 1), and each colony has overall reproductive efficiency *r*′.

Next, consider a subsequent mutation at the B locus, *b*, that acts in workers. The *b* allele, when expressed in a worker, causes it to become reproductive again, that is, to resist queen control. The *b* allele for worker reproduction can be either dominant, so that type *Bb* and type *bb* workers are reproductive, or recessive, so that only type *bb* workers are reproductive (Olejarz et al., 2015). If a colony contains only workers with the reproductive phenotype, then the fraction of queen‐derived males within the colony is *p*, and the overall reproductive efficiency of the colony is *r*. Thus, the *b* allele for worker reproduction essentially undoes the effects of the *a* allele for queen control.

What are the requirements for queen control to be evolutionarily stable against a mutation in workers that restores their reproduction? To answer this question for a dominant *b* allele, we turn to condition (53) in Olejarz et al. (2015), which is the condition, for any number of matings, *n*, for stability of a recessive mutation in workers that results in worker sterility: Setting *r*_1_ = *r*′ in condition (53) in Olejarz et al. (2015), this condition becomes

(3) $$\left[\frac{r^{\prime }}{r_{\frac{n-1}{n}}}-\frac{n\left(1-p_{\frac{n-1}{n}}\right)}{2}\right]\left[2\left(\frac{r^{\prime }}{r_{\frac{1}{2}}}\right)-1\right]>1,$$

In Condition (3), *r*_1/2_ is the colony reproductive efficiency when a fraction 1/2 of workers are reproductive, *r*_(*n*−1)/*n*_ is the colony reproductive efficiency when a fraction 1/*n* of workers are reproductive, and *p*_(*n*−1)/*n*_ is the fraction of queen‐derived males when a fraction 1/*n* of workers are reproductive. If Condition (3) is satisfied, then a subsequent dominant mutation, *b*, that acts in workers to restore their reproduction *cannot* invade a queen‐controlled population.

To further determine whether the dominant *b* allele cannot fix, we must also consider the condition directly after condition (34) in Olejarz et al. (2015), which is the condition, for any number of matings, *n*, for invasion of a recessive mutation in workers that results in worker sterility. Setting *p*_0_ = *p* and *r*_0_ = *r* in the condition directly after condition (34) in Olejarz et al. (2015), we obtain

(4) $$\frac{r_{\frac{1}{2n}}}{r}>\frac{2(2n-1)(2+n+np)}{2n^{2}\left(2+p+p_{\frac{1}{2n}}\right)+n\left(3+3p-2p_{\frac{1}{2n}}\right)-2\left(1+p\right)},$$

In Condition (4), *r*_1/(2*n*)_ is the colony reproductive efficiency when a fraction (2*n* − 1)/(2*n*) of workers are reproductive, and *p*_1/(2*n*)_ is the fraction of queen‐derived males when a fraction (2*n* − 1)/(2*n*) of workers are reproductive. If Condition (4) is satisfied, then a subsequent dominant mutation, *b*, that acts in workers to restore their reproduction *cannot* fix in the population.

Notice that Condition (3) depends on the parameters *r*_1/2_, *r*_(*n*−1)/*n*_, and *p*_(*n*−1)/*n*_, which are related to the effects of the *b* allele for worker reproduction. Also, notice that Condition (4) depends on the parameters *r*_1/(2*n*)_ and *p*_1/(2*n*)_, which are related to the effects of the *b* allele for worker reproduction. The properties of the particular dominant *b* allele for worker reproduction that is under consideration are therefore essential for determining whether the effects of the *a* allele for queen control can be undone by worker resistance.

There are many possible ways in which *p*_*z*_ and *r*_*z*_ in Conditions (3) and (4) could depend on *z*. To gain insight regarding the parameters *r*_1/2_, *r*_(*n*−1)/*n*_, *p*_(*n*−1)/*n*_, *r*_1/(2*n*)_, and *p*_1/(2*n*)_ in Conditions (3) and (4), we can consider the following simple case:

(5) $$\begin{array}{cc} p_{z} & =p+(1-p)z\\ r_{z} & =r+(r^{\prime }-r)z, \end{array}$$

For the parameter choices given by Equation (5), Condition (3) becomes

(6) $$\frac{r^{\prime }}{r}>\frac{4\left(1-p\right)+n\left(3+p\right)+\sqrt{{\left[4\left(1-p\right)+n\left(3+p\right)\right]}^{2}+4\left(1+p\right)\left[5+n+3\left(n-1\right)p\right]}}{2[5+n+3(n-1)p]},$$

Also for the parameter choices given by Equation (5), Condition (4) becomes

(7) $$\frac{r^{\prime }}{r}>\frac{3+4n+p}{3+p+2n(1+p)}.$$

To determine whether queen control is evolutionarily stable against a recessive *b* mutation in workers that restores their reproduction, we turn to the condition directly after condition (49) in Olejarz et al. (2015), which is the condition, for any number of matings, *n*, for stability of a dominant mutation in workers that results in worker sterility: Setting *r*_1_ = *r*′ in the condition directly after condition (49) in Olejarz et al. (2015), this condition becomes

(8) $$\frac{r^{\prime }}{r_{\frac{2n-1}{2n}}}>\frac{2+3n-np_{\frac{2n-1}{2n}}}{2(n+1)},$$

In Condition (8), *r*_(2*n*−1)/(2*n*)_ is the colony reproductive efficiency when a fraction 1/(2*n*) of workers are reproductive, and *p*_(2*n*−1)/(2*n*)_ is the fraction of queen‐derived males when a fraction 1/(2*n*) of workers are reproductive. If Condition (8) is satisfied, then a subsequent recessive mutation, *b*, that acts in workers to restore their reproduction *cannot* invade a queen‐controlled population.

To further determine whether the recessive *b* allele cannot fix, we must also consider condition (20) in Olejarz et al. (2015), which is the condition, for any number of matings, *n*, for invasion of a dominant mutation in workers that results in worker sterility. Setting *r*_0_ = *r* in condition (20) in Olejarz et al. (2015), we obtain

(9) $$\frac{r_{\frac{1}{2}}}{r}\left[1+p_{\frac{1}{2}}\left(\frac{r_{\frac{1}{n}}}{r}\right)\right]>2,$$

In Condition (9), *r*_1/*n*_ is the colony reproductive efficiency when a fraction (*n* − 1)/*n* of workers are reproductive, *r*_1/2_ is the colony reproductive efficiency when a fraction 1/2 of workers are reproductive, and *p*_1/2_ is the fraction of queen‐derived males when a fraction 1/2 of workers are reproductive. If Condition (9) is satisfied, then a subsequent recessive mutation, *b*, that acts in workers to restore their reproduction *cannot* fix in the population.

Notice that Condition (8) depends on the parameters *r*_(2*n*−1)/(2*n*)_ and *p*_(2*n*−1)/(2*n*)_, which are related to the effects of the *b* allele for worker reproduction. Also, notice that Condition (9) depends on the parameters *r*_1/*n*_, *r*_1/2_, and *p*_1/2_, which are related to the effects of the *b* allele for worker reproduction. The properties of the particular recessive *b* allele for worker reproduction that is under consideration are therefore essential for determining whether the effects of the *a* allele for queen control can be undone by worker resistance.

To gain insight regarding the parameters *r*_(2*n*−1)/(2*n*)_, *p*_(2*n*−1)/(2*n*)_, *r*_1/*n*_, *r*_1/2_, and *p*_1/2_ in Conditions (8) and (9), we can again consider the simple case given by Equation (5). For the parameter choices given by Equation (5), Condition (8) becomes

(10) $$\frac{r^{\prime }}{r}>\frac{5+4n-p}{5-p+2n(1+p)},$$

Also for the parameter choices given by Equation (5), Condition (9) becomes

(11) $$\frac{r^{\prime }}{r}>\frac{\sqrt{4n\left(5-p\right)\left(1+p\right)+4{\left(1+p\right)}^{2}+n^{2}{\left(3+p\right)}^{2}}-n\left(3+p\right)}{2(1+p)}.$$

**Figure 5:**
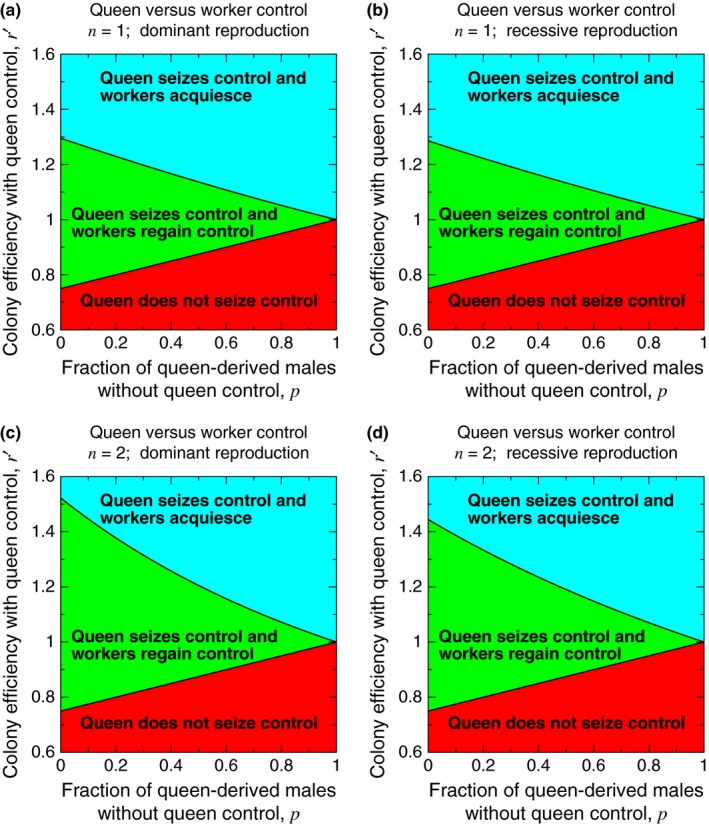
A mutation for queen control may or may not be in conflict with workers' evolutionary interests. We set *r* = 1 without loss of generality, and we assume that the queen‐control allele eliminates workers' reproduction. If the efficiency loss from queen control is too severe (corresponding to values of *r*′ in the red region), then queen control does not evolve (or it invades without fixing, and a subsequent mutation acting in workers causes them to become fully reproductive again). If the efficiency loss or gain from queen control is moderate (corresponding to values of *r*′ in the green region), then queen control evolves, but a subsequent mutation acting in workers causes them to become fully reproductive again. If the efficiency gain from queen control is sufficiently large (corresponding to values of *r*′ in the blue region), then queen control evolves, and workers subsequently acquiesce by remaining nonreproductive. The lower boundary is given by Condition (2), and the upper boundary is given by (a) Condition (6) for *n* = 1, (b) Condition (10) for *n* = 1, (c) Condition (6) for *n* = 2, and (d) Condition (10) for *n* = 2. For this plot, we use Equation (5), and we set *p*′ = 1 and *r* = 1

Figure 5 shows the evolutionary outcome of queen control for parameters *p* and *r*′. We set *r* = 1 without loss of generality. In each panel, the boundary between the lower, red region and the middle, green region is given by Condition (2). The boundary between the middle, green region and the upper, blue region is given by Condition (6) for *n* = 1 (Figure 5a), Condition (10) for *n* = 1 (Figure 5b), Condition (6) for *n* = 2 (Figure 5c), and Condition (10) for *n* = 2 (Figure 5d). For values (*p*, *r*′) in the lower, red region, the *a* mutation for queen control is unable to spread to fixation. For values (*p*, *r*′) in the middle, green region, the *a* mutation for queen control invades and is evolutionarily stable to noncontrol, but the subsequent *b* mutation for worker reproduction also invades and is evolutionarily stable, undoing the effects of queen control. For values (*p*, *r*′) in the upper, blue region, the *a* mutation for queen control invades and is evolutionarily stable to noncontrol, and the subsequent *b* mutation for worker reproduction is unable to invade, rendering queen control evolutionarily stable against counteraction by workers.

**Figure 6:**
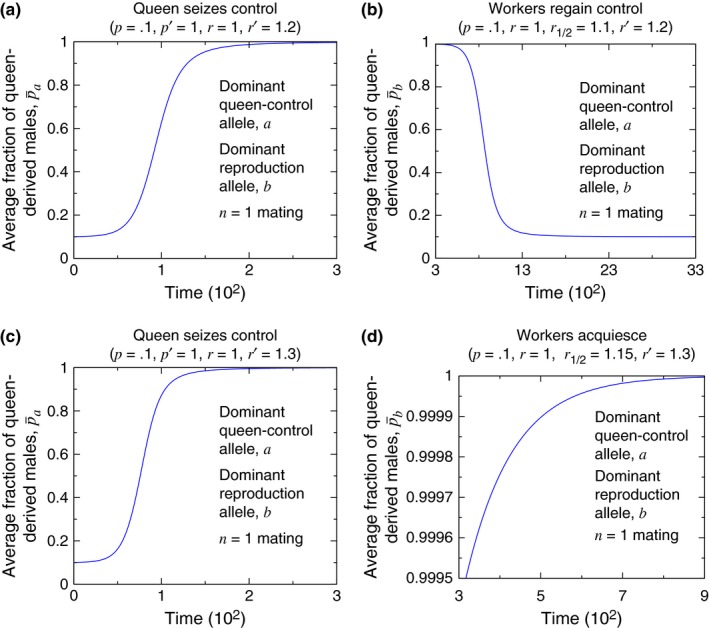
Simulations reveal the behaviors shown in Figure 5. It is possible that a mutation causing queen control evolves (a), and worker reproduction is subsequently restored (b). But if the efficiency gain due to queen control is large enough, then queen control evolves (c), and workers are unable to regain reproductive ability (d). (In (b) and (d), *r*_1/2_ denotes the colony efficiency when 1/2 of workers in the colony have the phenotype for worker reproduction. We follow the assumption for *r*_*z*_ in Equation (5) for determining the values of *r*_1/2_ = 1.1 (b) and *r*_1/2_ = 1.15 (d) for these simulations. The initial conditions are (a) *X*_*AA*, 0_ = 1 − 10^−3^ and *X*_*AA*, 1_ = 10^−3^, (b) *X*_*BB*, 0_ = 1 − 10^−3^ and *X*_*BB*, 1_ = 10^−3^, (c) *X*_*AA*, 0_ = 1 − 10^−3^ and *X*_*AA*, 1_ = 10^−3^, and (d) *X*_*BB*, 0_ = 1 − 10^−3^ and *X*_*BB*, 1_ = 10^−3^. For (b) and (d), we introduce the *b* allele for worker reproduction at time *t* = 300. For panels (a) and (c), ${\bar{p}}_{a}$ is given by Equation (12), while for panels (b) and (d), ${\bar{p}}_{b}$ is given by Equation (13). For calculating ${\bar{p}}_{b}$, *p*_1/2_ is given by the assumption for *p*_*z*_ in Equation (5).)

Corresponding simulations of the evolutionary dynamics are shown in Figure 6. In Figure 6, the quantity ${\bar{p}}_{a}$ or ${\bar{p}}_{b}$ that is plotted on the vertical axis is the average fraction of queen‐derived males in the population. As Figure 6(a,c) are for single mating (*n* = 1) and a dominant queen‐control allele, *a*, for those panels, we have

(12) $${\bar{p}}_{a}=\frac{pr\left(X_{AA,0}+X_{AA,1}\right)+p^{\prime }r^{\prime }\left(X_{Aa,0}+X_{Aa,1}+X_{aa,0}+X_{aa,1}\right)}{r\left(X_{AA,0}+X_{AA,1}\right)+r^{\prime }\left(X_{Aa,0}+X_{Aa,1}+X_{aa,0}+X_{aa,1}\right)},$$

Here, *X*_*AA*, 0_, *X*_*AA*, 1_, *X*_*Aa*, 0_, *X*_*Aa*, 1_, *X*_*aa*, 0_, and *X*_*aa*, 1_ are the frequencies of the six types of colonies in the population when considering the dynamics of the dominant queen‐control allele, *a*. As Figure 6(b,d) are for single mating (*n* = 1) and a dominant reproduction allele, *b*, for those panels, we have

(13) $${\bar{p}}_{b}=\frac{pr\left(X_{bb,1}+X_{bb,0}+X_{Bb,1}+X_{BB,1}\right)+p_{\frac{1}{2}}r_{\frac{1}{2}}X_{Bb,0}+p^{\prime }r^{\prime }X_{BB,0}}{r\left(X_{bb,1}+X_{bb,0}+X_{Bb,1}+X_{BB,1}\right)+r_{\frac{1}{2}}X_{Bb,0}+r^{\prime }X_{BB,0}},$$

Here, *X*_*BB*, 0_, *X*_*BB*, 1_, *X*_*Bb*, 0_, *X*_*Bb*, 1_, *X*_*bb*, 0_, and *X*_*bb*, 1_ are the frequencies of the six types of colonies in the population when considering the dynamics of the dominant reproduction allele, *b* (e.g., colonies headed by a type *BB* queen who has mated with a wild‐type *B* male are denoted *BB*, 0, while colonies headed by a type *BB* queen who has mated with a mutant *b* male are denoted *BB*, 1).

### 3.3 The effects of partial queen control

There is a subtlety, however. Figure 5 assumes that queen control can be easily undone by a single mutation in workers. This assumption is not necessarily true. A single mutation in a worker may not be sufficient to reverse the primer or releaser effects of a queen's complex pheromonal bouquet. The queen or dominant individual can also perform oophagy of worker‐laid eggs or physical aggression, and it is unclear whether a single mutation in a worker can enable her to overcome such behavioral dominance.

Thus, there is another important aspect to the question of evolutionary stability of queen control. In a wild‐type colony, the queen does not exert control over her workers' production of males. The queen produces a fraction *p* of males, and the colony's reproductive efficiency is *r*. In a mutant colony, it is possible—and perhaps most likely—that the queen only partially inhibits her workers' production of males. The queen produces a fraction *p*′ of males, where *p* < *p*′ < 1, and the colony's reproductive efficiency is *r*′. If a queen inhibits some—but not all—of her workers' parthenogenetic reproduction, then we term this phenomenon “partial queen control”. If there is a high genetic barrier against workers' resistance to partial queen control, then can partial queen control incentivize workers to become completely sterile (Charlesworth, 1978)?

Consider, again, an initially homogeneous population of colonies. Allele *A* is fixed at locus A, and allele *C* is fixed at locus C. Each colony's fraction of queen‐derived males is *p*, and each colony's overall reproductive efficiency is *r*. Suppose that a mutation at the A locus, *a*, acts in a queen, causing her to partially suppress her workers' production of drones. In colonies headed by partially controlling queens, a fraction *p*′ of males originate from the partially controlling queen, with *p* < *p*′ < 1, and the overall reproductive efficiency of the colony is *r*′. According to Conditions (1) and (2), if *r*′/*r* is sufficiently large, then the partially controlling queens will increase in frequency and fix in the population. Once the allele for partial queen control has fixed, a fraction *p*′ of each colony's male eggs originate from the queen, and each colony has overall reproductive efficiency *r*′.

Next, consider a subsequent mutation at the C locus, *c*, that acts in workers. The *c* allele changes a worker's phenotype, causing the mutant worker to become completely sterile. The *c* allele for worker sterility can be either recessive, so that only type *cc* workers are sterile, or dominant, so that type *Cc* and type *cc* workers are sterile (Olejarz et al., 2015). If a colony contains only workers with the phenotype for sterility, then the fraction of queen‐derived males within the colony is 1, and the overall reproductive efficiency of the colony is r*.

What are the requirements for partial queen control to enable the evolutionary success of a mutation in workers that renders them sterile? To answer this question for a recessive *c* allele, we turn to the condition directly after condition (34) in Olejarz et al. (2015), which is the condition, for any number of matings, *n*, for invasion of a recessive mutation in workers that causes worker sterility: Setting *p*_0_ = *p*′ and *r*_0_ = *r*′ in the condition directly after condition (34) in Olejarz et al. (2015), this condition becomes

(14) $$\frac{r_{\frac{1}{2n}}}{r^{\prime }}>\frac{2\left(2n-1\right)\left(2+n+np^{\prime }\right)}{2n^{2}\left(2+p^{\prime }+p_{\frac{1}{2n}}\right)+n\left(3+3p^{\prime }-2p_{\frac{1}{2n}}\right)-2\left(1+p^{\prime }\right)},$$

In Condition (14), *r*_1/(2*n*)_ is the colony reproductive efficiency when a fraction 1/(2*n*) of workers are sterile, and *p*_1/(2*n*)_ is the fraction of queen‐derived males when a fraction 1/(2*n*) of workers are sterile. If Condition (14) is satisfied, then a subsequent recessive mutation, *c*, that acts in workers to render them sterile invades a partially queen‐controlled population.

To further determine whether the recessive *c* allele can fix, we must also consider condition (53) in Olejarz et al. (2015), which is the condition, for any number of matings, *n*, for stability of a recessive mutation in workers that causes worker sterility. Setting *r*_1_ = r* in condition (53) in Olejarz et al. (2015), we obtain

(15) $$\left[\frac{r^{*}}{r_{\frac{n-1}{n}}}-\frac{n\left(1-p_{\frac{n-1}{n}}\right)}{2}\right]\left[2\left(\frac{r^{*}}{r_{\frac{1}{2}}}\right)-1\right]>1,$$

In Condition (15), *r*_1/2_ is the colony reproductive efficiency when a fraction 1/2 of workers are sterile, *r*_(*n*−1)/*n*_ is the colony reproductive efficiency when a fraction (*n* − 1)/*n* of workers are sterile, and *p*_(*n*−1)/*n*_ is the fraction of queen‐derived males when a fraction (*n* − 1)/*n* of workers are sterile. If Condition (15) is satisfied, then a subsequent recessive mutation, *c*, that acts in workers to render them sterile is evolutionarily stable.

Notice that Condition (14) depends on the parameters *r*_1/(2*n*)_ and *p*_1/(2*n*)_, which are related to the effects of the *c* allele for worker sterility. Also, notice that Condition (15) depends on the parameters r*, *r*_1/2_, *r*_(*n*−1)/*n*_, and *p*_(*n*−1)/*n*_, which are related to the effects of the *c* allele for worker sterility. The properties of the particular recessive *c* allele for worker sterility that is under consideration are therefore essential for determining whether the *a* allele for partial queen control can facilitate the evolution of complete worker sterility.

There are many possible ways in which *p*_*z*_ and *r*_*z*_ in Conditions (14) and (15) could depend on *z*. To gain insight, regarding the parameters *r*_1/(2*n*)_, *p*_1/(2*n*)_, *r*_1/2_, *r*_(*n*−1)/*n*_, and *p*_(*n*−1)/*n*_ in Conditions (14) and (15), we can consider the following simple case:

(16) $$\begin{array}{cc} p_{z} & =p^{\prime }+\left(1-p^{\prime }\right)z\\ r_{z} & =r^{\prime }+\left(r^{*}-r^{\prime }\right)z, \end{array}$$

For the parameter choices given by Equation (16), Condition (14) becomes

(17) $$\frac{r^{*}}{r^{\prime }}>\frac{3+4n+p^{\prime }}{3+p^{\prime }+2n\left(1+p^{\prime }\right)},$$

Also for the parameter choices given by Equation (16), Condition (15) becomes

(18) $$\begin{array}{cc} & \frac{r^{*}}{r^{\prime }}>\\ & \frac{4\left(1-p^{\prime }\right)+n\left(3+p^{\prime }\right)+\sqrt{{\left[4\left(1-p^{\prime }\right)+n\left(3+p^{\prime }\right)\right]}^{2}+4\left(1+p^{\prime }\right)\left[5+n+3\left(n-1\right)p^{\prime }\right]}}{2[5+n+3\left(n-1\right)p^{\prime }]}. \end{array}$$

To determine whether partial queen control can enable the evolutionary success of a dominant *c* mutation in workers that renders them sterile, we turn to condition (20) in Olejarz et al. (2015), which is the condition, for any number of matings, *n*, for invasion of a dominant mutation in workers that results in worker sterility: Setting *r*_0_ = *r*′ in condition (20) in Olejarz et al. (2015), this condition becomes

(19) $$\frac{r_{\frac{1}{2}}}{r^{\prime }}\left[1+p_{\frac{1}{2}}\left(\frac{r_{\frac{1}{n}}}{r^{\prime }}\right)\right]>2,$$

In Condition (19), *r*_1/*n*_ is the colony reproductive efficiency when a fraction 1/*n* of workers are sterile, *r*_1/2_ is the colony reproductive efficiency when a fraction 1/2 of workers are sterile, and *p*_1/2_ is the fraction of queen‐derived males when a fraction 1/2 of workers are sterile. If Condition (19) is satisfied, then a subsequent dominant mutation, *c*, that acts in workers to render them sterile invades a partially queen‐controlled population.

To further determine whether the dominant *c* allele can fix, we must also consider the condition directly after condition (49) in Olejarz et al. (2015), which is the condition, for any number of matings, *n*, for stability of a dominant mutation in workers that causes worker sterility. Setting *r*_1_ = r* in the condition directly after condition (49) in Olejarz et al. (2015), we obtain

(20) $$\frac{r^{*}}{r_{\frac{2n-1}{2n}}}>\frac{2+3n-np_{\frac{2n-1}{2n}}}{2(n+1)},$$

In Condition (20), *r*_(2*n*−1)/(2*n*)_ is the colony reproductive efficiency when a fraction (2*n* − 1)/(2*n*) of workers are sterile, and *p*_(2*n*−1)/(2*n*)_ is the fraction of queen‐derived males when a fraction (2*n* − 1)/(2*n*) of workers are sterile. If Condition (20) is satisfied, then a subsequent dominant mutation, *c*, that acts in workers to render them sterile is evolutionarily stable.

Notice that Condition (19) depends on the parameters *r*_1/*n*_, *r*_1/2_, and *p*_1/2_, which are related to the effects of the *c* allele for worker sterility. Also, notice that Condition (20) depends on the parameters r*, *r*_(2*n*−1)/(2*n*)_, and *p*_(2*n*−1)/(2*n*)_, which are related to the effects of the *c* allele for worker sterility. The properties of the particular dominant *c* allele for worker sterility that is under consideration are therefore essential for determining whether the *a* allele for partial queen control can facilitate the evolution of complete worker sterility.

To gain insight, regarding the parameters *r*_1/*n*_, *r*_1/2_, *p*_1/2_, *r*_(2*n*−1)/(2*n*)_, and *p*_(2*n*−1)/(2*n*)_ in Conditions (19) and (20), we can again consider the simple case given by Equation (16).

For the parameter choices given by Equation (16), Condition (19) becomes

(21) $$\frac{r^{*}}{r^{\prime }}>\frac{\sqrt{4n\left(5-p^{\prime }\right)\left(1+p^{\prime }\right)+4{\left(1+p^{\prime }\right)}^{2}+n^{2}{\left(3+p^{\prime }\right)}^{2}}-n\left(3+p^{\prime }\right)}{2(1+p^{\prime })},$$

Also for the parameter choices given by Equation (16), Condition (20) becomes

(22) $$\frac{r^{*}}{r^{\prime }}>\frac{5+4n-p^{\prime }}{5-p^{\prime }+2n\left(1+p^{\prime }\right)}.$$

**Figure 7:**
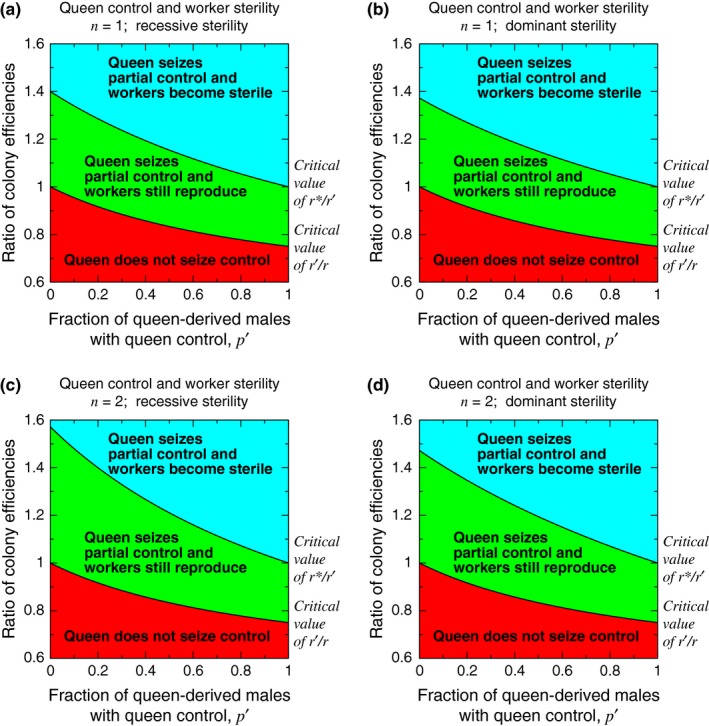
A mutation for queen control may or may not induce the subsequent evolution of worker sterility. Initially, assume that workers are responsible for all male production (*p* = 0). A mutation in queens then causes them to seize partial control of male production (0 < *p*′ < 1). More powerful queen control (i.e., mutations causing larger values of *p*′) can evolve more easily, as the critical value of *r*′/*r* decreases with *p*′. But more powerful queen control also lowers the critical value of r*/*r*′ for a subsequent mutation, acting in workers, to render them sterile. The lower boundary is given by Condition (2), and the upper boundary is given by (a) Condition (17) for *n* = 1, (b) Condition (21) for *n* = 1, (c) Condition (17) for *n* = 2, and (d) Condition (21) for *n* = 2. For this plot, we use Equation (16), and we set *p* = 0. (If we considered *p* > 0 instead, then, when plotted between *p* < *p*′ < 1 on the horizontal axis, this figure would look qualitatively the same, except that the middle, green region would be smaller.)

Figure 7 shows how partial queen control can facilitate complete worker sterility. In each panel, the boundary between the lower, red region and the middle, green region is given by Condition (2). For values (*p*′, *r*′/*r*) in the lower, red region, the queen does not seize partial control. For values (*p*′, *r*′/*r*) in the middle, green region or the upper, blue region, the queen seizes partial control, and the workers may or may not become sterile. The boundary between the middle, green region and the upper, blue region is given by Condition (17) for *n* = 1 (Figure 7a), Condition (21) for *n* = 1 (Figure 7b), Condition (17) for *n* = 2 (Figure 7c), and Condition (21) for *n* = 2 (Figure 7d). This boundary determines whether workers become sterile after the queen has seized partial control of male production. Suppose that the queen seizes partial control of male production. For values (*p*′, r*/*r*′) in the lower, red region or the middle, green region, the *c* mutation for worker sterility does not invade. For values (*p*′, r*/*r*′) in the upper, blue region, the *c* mutation for worker sterility invades and is evolutionarily stable, rendering workers totally nonreproductive.

**Figure 8:**
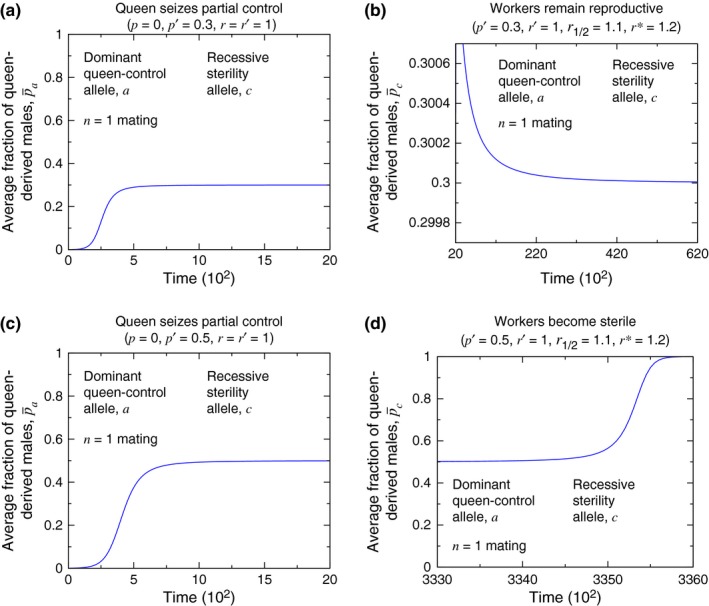
Simulations reveal the behaviors shown in Figure 7. If queens seize a small amount of control over male production (a), then a subsequent mutation, acting in workers, does not cause them to become sterile (b). If queens seize a large amount of control over male production (c), then a subsequent mutation, acting in workers, causes them to become sterile (d). Thus, queen control can facilitate the formation of a sterile worker caste. (In (b) and (d), *r*_1/2_ denotes the colony efficiency when 1/2 of workers in the colony have the phenotype for worker sterility. We follow the assumption for *r*_*z*_ in Equation (16) for determining the value of *r*_1/2_ = 1.1 for these simulations. The initial conditions are (a) *X*_*AA*, 0_ = 1 − 10^−3^ and *X*_*AA*, 1_ = 10^−3^, (b) *X*_*CC*, 0_ = 1 − 10^−1^ and *X*_*CC*, 1_ = 10^−1^, (c) *X*_*AA*, 0_ = 1 − 10^−3^ and *X*_*AA*, 1_ = 10^−3^, and (d) *X*_*CC*, 0_ = 1 − 10^−3^ and *X*_*CC*, 1_ = 10^−3^. For (b) and (d), we introduce the *c* allele for worker sterility at time *t* = 2,000. For panels (a) and (c), ${\bar{p}}_{a}$ is given by Equation (12), while for panels (b) and (d), ${\bar{p}}_{c}$ is given by Equation (23). For calculating ${\bar{p}}_{c}$, *p*_1/2_ is given by the assumption for *p*_*z*_ in Equation (16).)

Corresponding simulations of the evolutionary dynamics are shown in Figure 8. In Figure 8, the quantity ${\bar{p}}_{a}$ or ${\bar{p}}_{c}$ that is plotted on the vertical axis is the average fraction of queen‐derived males in the population. As Figure 8(a,c) are for single mating (*n* = 1) and a dominant queen‐control allele, *a*, for those panels, we use Equation (12). As Figure 8(b,d) are for single mating (*n* = 1) and a recessive sterility allele, *c*, for those panels, we have

(23) $${\bar{p}}_{c}=\frac{p^{\prime }r^{\prime }\left(X_{CC,0}+X_{CC,1}+X_{Cc,0}+X_{cc,0}\right)+p_{\frac{1}{2}}r_{\frac{1}{2}}X_{Cc,1}+r^{*}X_{cc,1}}{r^{\prime }\left(X_{CC,0}+X_{CC,1}+X_{Cc,0}+X_{cc,0}\right)+r_{\frac{1}{2}}X_{Cc,1}+r^{*}X_{cc,1}},$$

Here, *X*_*CC*, 0_, *X*_*CC*, 1_, *X*_*Cc*, 0_, *X*_*Cc*, 1_, *X*_*cc*, 0_, and *X*_*cc*, 1_ are the frequencies of the six types of colonies in the population when considering the dynamics of the recessive sterility allele, *c* (e.g., colonies headed by a type *CC* queen who has mated with a wild‐type *C* male are denoted *CC*, 0, while colonies headed by a type *CC* queen who has mated with a mutant *c* male are denoted *CC*, 1).

## 4 DISCUSSION

We have studied, in a haplodiploid population‐genetic model of a social Hymenopteran, the conditions for invasion and fixation of genes that act in queens to suppress worker reproduction. We have also studied the conditions under which selection subsequently favors genes that act in workers to resist queen control. There always exist regions of parameter space where queen control can invade and fix, but where worker suppression of queen control is subsequently selected for. In these cases, queen control can be thought of as coercive (i.e., against workers' evolutionary interests). There also always exist regions of parameter space where queen control invades and fixes, and where the conditions for worker acquiescence are satisfied—here, evolved queen control can be thought of as honest signaling (i.e., in workers' evolutionary interests). We have thus shown that, within the same simple setup, both coercive control and control via honest signaling are possible.

The crucial consideration in our analysis is how the establishment of queen control changes two colony‐level empirical parameters: the colony's overall reproductive efficiency (to *r*′, from a value of *r* in colonies without queen control) and the proportion of males that are produced by the queen (to *p*′, from a value of *p* in colonies without queen control). The efficiency threshold, *r*′/*r*, needed for a queen‐control allele to evolve and fix, given by Condition (2), decreases with the strength of queen control (i.e., the amount by which *p*′ exceeds *p*). In other words, for all values *p*′ > *p*, queen control can evolve and fix even if it results in the colony being less productive at making new individuals. However, the efficiency threshold, *r*′/*r*, needed for a queen‐control allele to be stable to counteraction by workers, given by Conditions (6) or (10), increases with the strength of queen control. In other words, for all values *p*′ > *p*, queen control cannot be evolutionarily stable against counteraction by workers unless it increases the productivity of the colony.

This result has significant implications for the evolutionary history of queen control in the social insects. A mutation that acts in queens, causing them to increase the fraction of queen‐derived offspring, can invade if it does not reduce colony efficiency by too much, but will be unstable with respect to the invasion of worker resistance if it does not sufficiently increase colony efficiency. Therefore, if *r*′/*r* is sufficiently close to 1, then queen control fixes but is promptly suppressed by worker resistance. But such mutations of weak phenotypic effect on colony efficiency were likely common in the evolutionary history of social insects (Charlesworth, 1978; Geritz, Kisdi, Meszena, & Metz, 1998; Olejarz et al., 2016). It follows that continual arms‐race evolution—with the queen seizing increased control over male production, and the workers subsequently regaining control—is likely to have been a natural state of affairs in the evolutionary development of advanced forms of sociality.

Although this kind of pattern is well‐known from “battleground” models of parent–offspring conflict (Godfray, 1995; Trivers, 1974; Yamamura & Higashi, 1992), this result is interesting in light of the continuing empirical debate over whether queen control represents coercion or honest signaling. Many recent works have expressed disfavor toward the coercion interpretation (Chapuisat, 2014; Holman, 2010; Keller & Nonacs, 1993; Oi, van Zweden, et al., 2015; Peso et al., 2015; van Zweden, Bonckaert, Wenseleers, & d'Ettorre, 2013). Yet, regardless of the specific steps that ultimately led to eusociality, the existence of such a queen–worker arms‐race conflict over the evolutionary history of the eusocial Hymenoptera is strongly predicted by our findings. On the empirical and experimental side, research is underway on the chemical characteristics of queen‐emitted pheromones that induce specific primer or releaser effects on workers (Bello, McElfresh, & Millar, 2015; Eliyahu, Ross, Haight, Keller, & Liebig, 2011; Sharma et al., 2015; Smith, Hӧlldobler, & Liebig, 2012; Van Oystaeyen et al., 2014; Wagner et al., 1998; Yew & Chung, 2015; Zhou et al., 2015) and on the molecular mechanisms and gene networks behind reproductive regulation (Fischman, Woodard, & Robinson, 2011; Khila & Abouheif, 2008, 2010; Kocher, Ayroles, Stone, & Grozinger, 2010; Mullen, Daley, Backx, & Thompson, 2014; Rehan, Berens, & Toth, 2014; Rehan & Toth, 2015; Ronai, Oldroyd, et al., 2016a; Ronai et al., 2016c; Thompson, Yockey, Lim, & Oldroyd, 2007; Toth et al., 2014). Such programs promise to elucidate the precise mechanisms by which the predicted queen–worker arms race over male production could manifest.

Intriguingly, the queen's continual efforts to suppress her workers' reproduction are not always necessarily countered with worker resistance. The efficiency increase, *r*′/*r*, needed for a queen‐control allele to be stable to counteraction by workers, given by Conditions (6) or (10), increases with the strength of queen control (i.e., the amount by which *p*′ exceeds *p*). But the efficiency increase, r*/*r*′, needed for a subsequent allele, acting in workers, to induce their sterility, given by Conditions (17) or (21), decreases with the strength of queen control (i.e., the magnitude of *p*′). Thus, stronger queen control is more susceptible to worker resistance, but it also more easily selects for worker nonreproduction.

Moreover, in our analysis, colony efficiencies with and without queen control are treated as static parameters. However, because queen control directly limits the workers' contribution to the production of drones, it makes it beneficial for workers instead to invest their resources in colony maintenance tasks (Wenseleers, Hart, & Ratnieks, 2004; Wenseleers & Ratnieks, 2006). Therefore, colony efficiency could change if the evolution of queen‐induced worker sterility is followed by the evolution of more efficient helping by workers (González‐Forero, 2014, 2015). Under this scenario, it is possible that queen control establishes in a system where worker resistance is initially under positive selection—Conditions (6) and (10) do not hold—but that subsequent efficiency gains by the now‐sterile worker caste increase *r*′ sufficiently that Conditions (6) and (10) come to hold, so that worker resistance is no longer selected for.

## CONFLICT OF INTEREST

None declared.

## AUTHOR CONTRIBUTIONS

All authors contributed to this work.
