# Supplementary material for: The evolution of queen control over worker reproduction in the social Hymenoptera
Source: Ecol Evol. 2017 Sep 10;7(20):8427–41. doi: 10.1002/ece3.3324 (PMC5648666; doi:10.1002/ece3.3324)
Supplement: Supplementary file 1 [file ECE3-7-8427-s001.PDF]

A Supporting Information

In this Supporting Information, we derive evolutionary invasion and stability conditions for queen control of worker reproduction. The mathematical structure of our model is identical to the model featured in Olejarz et al. (2016). There are three types of females: AA, Aa, and aa. There are two types of males: A and a. The numbers of the three types of unfertilized females are denoted by x_{AA}, x_{Aa}, and x_{aa}. The numbers of the two types of males are denoted by y_A and y_a. Each colony is headed by a single, reproductive female. The $3(n+1)$ types of colonies are denoted by AA, m; Aa, m; and aa, m, where m is the number of the queen's matings that were with type a males. We naturally have that $0 \leq m \leq n$. The frequencies of the $3(n+1)$ types of colonies are denoted by $X_{AA,m}$, $X_{Aa,m}$, and $X_{aa,m}$. The mating events are shown in Figure 1(a). The reproduction events are shown in Figure 1(b).

The evolutionary dynamics are specified by the following equations:

$$\begin{aligned} \dot{X}_{AA,m} &= \frac{dX_{AA,m}}{dt} = \binom{n}{m} x_{AA} y_A^{n-m} y_a^m - \phi X_{AA,m} \\ \dot{X}_{Aa,m} &= \frac{dX_{Aa,m}}{dt} = \binom{n}{m} x_{Aa} y_A^{n-m} y_a^m - \phi X_{Aa,m} \\ \dot{X}_{aa,m} &= \frac{dX_{aa,m}}{dt} = \binom{n}{m} x_{aa} y_A^{n-m} y_a^m - \phi X_{aa,m} \end{aligned} \tag{24}$$

We impose the following density constraint:

$$\sum_{m=0}^{n} (X_{AA,m} + X_{Aa,m} + X_{aa,m}) = 1 \tag{25}$$

To enforce Equation (25), we set

$$\phi = (x_{AA} + x_{Aa} + x_{aa})(y_A + y_a)^n \tag{26}$$

A.1 Invasion of a Dominant Queen-Control Allele

We focus on the evolution of the colony frequencies. Using Figure 1(b), we write the number of each type of reproductive of a colony (x_{AA}, x_{Aa}, x_{aa}, y_A, and y_a) as follows:

$$\begin{aligned}
x_{AA} &= \sum_{m=0}^{n}\left[\frac{n-m}{n}rX_{AA,m} + \frac{n-m}{2n}r'X_{Aa,m}\right] \\
x_{Aa} &= \sum_{m=0}^{n}\left[\frac{m}{n}rX_{AA,m} + \frac{1}{2}r'X_{Aa,m} + \frac{n-m}{n}r'X_{aa,m}\right] \\
x_{aa} &= \sum_{m=0}^{n}\left[\frac{m}{2n}r'X_{Aa,m} + \frac{m}{n}r'X_{aa,m}\right] \\
y_A &= \sum_{m=0}^{n}\left[\frac{2n-m(1-p)}{2n}rX_{AA,m} + \frac{3n-2m+(2m-n)p'}{4n}r'X_{Aa,m}\right. \\
&\quad \left. + \frac{(n-m)(1-p')}{2n}r'X_{aa,m}\right] \\
y_a &= \sum_{m=0}^{n}\left[\frac{m(1-p)}{2n}rX_{AA,m} + \frac{n+2m+(n-2m)p'}{4n}r'X_{Aa,m}\right. \\
&\quad \left. + \frac{n+m+(n-m)p'}{2n}r'X_{aa,m}\right]
\end{aligned} \tag{27}$$

Among Equations (24), the three equations that are relevant for considering invasion of a rare, dominant a allele are

$$\begin{aligned}
\dot{X}_{AA,0} &= x_{AA}y_A^n - \phi X_{AA,0} \\
\dot{X}_{AA,1} &= nx_{AA}y_A^{n-1}y_a - \phi X_{AA,1} \\
\dot{X}_{Aa,0} &= x_{Aa}y_A^n - \phi X_{Aa,0}
\end{aligned} \tag{28}$$

If a small amount of the a allele is introduced into the population, then shortly after the perturbation, the colony frequencies have the following form (with $\epsilon \ll 1$):

$$\begin{aligned} X_{AA,0} &= 1 \quad - \epsilon\delta^{(1)}_{AA,0} - \mathcal{O}(\epsilon^2) \\ X_{AA,1} &= \qquad + \epsilon\delta^{(1)}_{AA,1} + \mathcal{O}(\epsilon^2) \\ X_{Aa,0} &= \qquad + \epsilon\delta^{(1)}_{Aa,0} + \mathcal{O}(\epsilon^2) \end{aligned} \tag{29}$$

Using Equations (29), the density constraint, Equation (25), takes the following form at $\mathcal{O}(\epsilon)$:

$$\delta^{(1)}_{AA,0} = \delta^{(1)}_{AA,1} + \delta^{(1)}_{Aa,0} \tag{30}$$

We substitute Equations (26), (27), (29), and (30) into Equations (28). We find that the condition for evolution of queen control is that the dominant eigenvalue of the Jacobian matrix in the following equation is greater than zero:

$$\begin{pmatrix} \dot{\delta}^{(1)}_{AA,1} \\ \dot{\delta}^{(1)}_{Aa,0} \end{pmatrix} = r^n \begin{pmatrix} \frac{-(1+p)r}{2} & \frac{(1+p')nr'}{4} \\ \frac{r}{n} & \frac{r'-2r}{2} \end{pmatrix} \begin{pmatrix} \delta^{(1)}_{AA,1} \\ \delta^{(1)}_{Aa,0} \end{pmatrix}$$

The dominant allele for queen control of worker reproduction increases in frequency if

$$\frac{r'}{r} > \frac{2(1+p)}{2+p+p'} \tag{31}$$

Condition (31) can be written more intuitively:

$$\frac{r'}{r} > 1 - \frac{p'-p}{2+p+p'}$$

A.1.1 Alternative Derivation

In an alternative treatment, we can consider evolution in discrete time. Consider a small amount of the mutant allele, a, in the population. $x_{Aa}(T)$ denotes the abundance of heterozygous mutant females in generation T, and $y_a(T)$ denotes the abundance of mutant males in generation T. Assuming that each new generation consists only of offspring from the previous generation, what are the abundances of x_{Aa} and y_a in the next generation, $T+1$?

Consider the following reproduction events. Aa females mate with wild-type A males at rate 1 to make $Aa, 0$ colonies, and $Aa, 0$ colonies make new Aa females at rate $r'/2$. a males mate with wild-type AA females at rate n to make $AA, 1$ colonies, and $AA, 1$ colonies make new Aa females at rate r/n. Aa females mate with wild-type A males at rate 1 to make $Aa, 0$ colonies, and $Aa, 0$ colonies make new a males at rate $p'r'/2 + (1-p')r'/4 = (1+p')r'/4$. a males mate with wild-type AA females at rate n to make $AA, 1$ colonies, and $AA, 1$ colonies make new a males at rate $(1-p)r/(2n)$. These reproduction events can be summarized as:

$$\begin{pmatrix} x_{Aa}(T+1) \\ y_a(T+1) \end{pmatrix} = \begin{pmatrix} \frac{r'}{2} & r \\ \frac{(1+p')r'}{4} & \frac{(1-p)r}{2} \end{pmatrix} \begin{pmatrix} x_{Aa}(T) \\ y_a(T) \end{pmatrix} \tag{32}$$

The condition for invasion of queen control is that the dominant eigenvalue of the matrix in Equation (32) is greater than r. Performing this calculation gives us Condition (31).

A.2 Invasion of a Recessive Queen-Control Allele

We again focus on evolution of the colony frequencies. Using Figure 1(b), we write the number of each type of reproductive of a colony (x_{AA}, x_{Aa}, x_{aa}, y_A, and y_a) as follows:

$$\begin{aligned}
x_{AA} &= \sum_{m=0}^{n} \left[\frac{n-m}{n} r X_{AA,m} + \frac{n-m}{2n} r X_{Aa,m} \right] \\
x_{Aa} &= \sum_{m=0}^{n} \left[\frac{m}{n} r X_{AA,m} + \frac{1}{2} r X_{Aa,m} + \frac{n-m}{n} r' X_{aa,m} \right] \\
x_{aa} &= \sum_{m=0}^{n} \left[\frac{m}{2n} r X_{Aa,m} + \frac{m}{n} r' X_{aa,m} \right] \\
y_A &= \sum_{m=0}^{n} \left[\frac{2n - m(1-p)}{2n} r X_{AA,m} + \frac{3n - 2m + (2m-n)p}{4n} r X_{Aa,m} \right. \\
&\quad \left. + \frac{(n-m)(1-p')}{2n} r' X_{aa,m} \right] \\
y_a &= \sum_{m=0}^{n} \left[\frac{m(1-p)}{2n} r X_{AA,m} + \frac{n + 2m + (n-2m)p}{4n} r X_{Aa,m} \right. \\
&\quad \left. + \frac{n + m + (n-m)p'}{2n} r' X_{aa,m} \right]
\end{aligned} \tag{33}$$

Among Equations (24), the six equations that are relevant for considering invasion of a rare, recessive a allele are

$$\begin{aligned}
\dot{X}_{AA,0} &= x_{AA} y_A^n - \phi X_{AA,0} \\
\dot{X}_{AA,1} &= n x_{AA} y_A^{n-1} y_a - \phi X_{AA,1} \\
\dot{X}_{Aa,0} &= x_{Aa} y_A^n - \phi X_{Aa,0} \\
\dot{X}_{AA,2} &= \frac{n(n-1)}{2} x_{AA} y_A^{n-2} y_a^2 - \phi X_{AA,2} \\
\dot{X}_{Aa,1} &= n x_{Aa} y_A^{n-1} y_a - \phi X_{Aa,1} \\
\dot{X}_{aa,0} &= x_{aa} y_A^n - \phi X_{aa,0}
\end{aligned} \tag{34}$$

If a small amount of the a allele is introduced into the population, then shortly after the perturbation, the colony frequencies have the following form (with $\epsilon \ll 1$):

$$\begin{aligned}
X_{AA,0} &= 1 \quad -\epsilon\delta^{(1)}_{AA,0} - \epsilon^2\delta^{(2)}_{AA,0} \quad -\mathcal{O}(\epsilon^3) \\
X_{AA,1} &= \quad +\epsilon\delta^{(1)}_{AA,1} + \epsilon^2\delta^{(2)}_{AA,1} \quad +\mathcal{O}(\epsilon^3) \\
X_{Aa,0} &= \quad +\epsilon\delta^{(1)}_{Aa,0} + \epsilon^2\delta^{(2)}_{Aa,0} \quad +\mathcal{O}(\epsilon^3) \\
X_{AA,2} &= \quad + \epsilon^2\delta^{(2)}_{AA,2} \quad +\mathcal{O}(\epsilon^3) \\
X_{Aa,1} &= \quad + \epsilon^2\delta^{(2)}_{Aa,1} \quad +\mathcal{O}(\epsilon^3) \\
X_{aa,0} &= \quad + \epsilon^2\delta^{(2)}_{aa,0} \quad +\mathcal{O}(\epsilon^3)
\end{aligned} \tag{35}$$

Equations (35), together with the density constraint, Equation (25), yield Equation (30) at $\mathcal{O}(\epsilon)$. At $\mathcal{O}(\epsilon^2)$, the density constraint, Equation (25), takes the following form:

$$\delta^{(2)}_{AA,0} = \delta^{(2)}_{AA,1} + \delta^{(2)}_{Aa,0} + \delta^{(2)}_{AA,2} + \delta^{(2)}_{Aa,1} + \delta^{(2)}_{aa,0} \tag{36}$$

We substitute Equations (26), (33), (35), and (30) into Equations (34). At $\mathcal{O}(\epsilon)$, we have

$$\begin{pmatrix} \dot{\delta}^{(1)}_{AA,1} \\ \dot{\delta}^{(1)}_{Aa,0} \end{pmatrix} = r^{n+1} \begin{pmatrix} \frac{-(1+p)}{2} & \frac{(1+p)n}{4} \\ \frac{1}{n} & \frac{-1}{2} \end{pmatrix} \begin{pmatrix} \delta^{(1)}_{AA,1} \\ \delta^{(1)}_{Aa,0} \end{pmatrix} \tag{37}$$

The dominant eigenvalue of the matrix in (37) is zero, and the corresponding eigenvector is

$$\begin{pmatrix} \delta^{(1)}_{AA,1} \\ \delta^{(1)}_{Aa,0} \end{pmatrix} = \frac{\delta^{(1)}_{AA,0}}{n+2} \begin{pmatrix} n \\ 2 \end{pmatrix} \tag{38}$$

We therefore use (38) in the following calculations.

We then substitute Equations (26), (33), (35), (38), and (36) into Equations (34). At

$\mathcal{O}(\epsilon^2)$, we have

$$\begin{aligned} -\dot{\delta}^{(2)}_{AA,0} r^{-(n+1)} = &\frac{2-n-np}{4n}\left(-2\delta^{(2)}_{AA,1} + n\delta^{(2)}_{Aa,0}\right) \\ &+ \frac{-2+np}{n}\delta^{(2)}_{AA,2} \\ &- \frac{n^2+2+n(n-2)p}{4n}\delta^{(2)}_{Aa,1} \\ &+ \frac{2-(2+n+np')r'r^{-1}}{2}\delta^{(2)}_{aa,0} \\ &+ \frac{n(n+3)}{2(n+2)^2}\left[\delta^{(1)}_{AA,0}\right]^2 \end{aligned} \tag{39}$$

We also have

$$\begin{aligned} \dot{\delta}^{(2)}_{AA,1} r^{-(n+1)} = &\frac{1+p}{4}\left(-2\delta^{(2)}_{AA,1} + n\delta^{(2)}_{Aa,0}\right) \\ &+ (1-p)\delta^{(2)}_{AA,2} \\ &+ \frac{n+2+(n-2)p}{4}\delta^{(2)}_{Aa,1} \\ &+ \frac{(1+p')nr'r^{-1}}{2}\delta^{(2)}_{aa,0} \\ &- \frac{n(n+1)}{(n+2)^2}\left[\delta^{(1)}_{AA,0}\right]^2 \\ \dot{\delta}^{(2)}_{Aa,0} r^{-(n+1)} = &\frac{-1}{2n}\left(-2\delta^{(2)}_{AA,1} + n\delta^{(2)}_{Aa,0}\right) \\ &+ \frac{2}{n}\delta^{(2)}_{AA,2} \\ &+ \frac{1}{2}\delta^{(2)}_{Aa,1} \\ &+ r'r^{-1}\delta^{(2)}_{aa,0} \\ &- \frac{2n}{(n+2)^2}\left[\delta^{(1)}_{AA,0}\right]^2 \\ \dot{\delta}^{(2)}_{AA,2} r^{-(n+1)} = &-\delta^{(2)}_{AA,2} + \frac{n(n-1)}{2(n+2)^2}\left[\delta^{(1)}_{AA,0}\right]^2 \\ \dot{\delta}^{(2)}_{Aa,1} r^{-(n+1)} = &-\delta^{(2)}_{Aa,1} + \frac{2n}{(n+2)^2}\left[\delta^{(1)}_{AA,0}\right]^2 \\ \dot{\delta}^{(2)}_{aa,0} r^{-(n+1)} = &-\delta^{(2)}_{aa,0} + \frac{1}{2n}\delta^{(2)}_{Aa,1} \end{aligned}$$

Integrating the equation for $\dot{\delta}^{(2)}_{AA,2}$, we get

$$\delta^{(2)}_{AA,2} = \frac{n(n-1)}{2(n+2)^2}\left[\delta^{(1)}_{AA,0}\right]^2\left[1-\exp(-r^{n+1}t)\right] \tag{40}$$

Integrating the equation for $\dot{\delta}^{(2)}_{Aa,1}$, we get

$$\delta^{(2)}_{Aa,1} = \frac{2n}{(n+2)^2}\left[\delta^{(1)}_{AA,0}\right]^2 \left[1 - \exp(-r^{n+1}t)\right] \tag{41}$$

Using the solution for $\delta^{(2)}_{Aa,1}$ to solve for $\delta^{(2)}_{aa,0}$, we get

$$\delta^{(2)}_{aa,0} = \frac{1}{(n+2)^2}\left[\delta^{(1)}_{AA,0}\right]^2 \left[1 - (1 + r^{n+1}t)\exp(-r^{n+1}t)\right] \tag{42}$$

The equations for $\dot{\delta}^{(2)}_{AA,1}$ and $\dot{\delta}^{(2)}_{Aa,0}$ can be manipulated to yield

$$\begin{aligned} r^{-(n+1)}\frac{d}{dt}\left(-2\delta^{(2)}_{AA,1} + n\delta^{(2)}_{Aa,0}\right) = {} & \frac{-(2+p)}{2}\left(-2\delta^{(2)}_{AA,1} + n\delta^{(2)}_{Aa,0}\right) \\ & + 2p\delta^{(2)}_{AA,2} \\ & - \frac{2+(n-2)p}{2}\delta^{(2)}_{Aa,1} \\ & - np'r'r^{-1}\delta^{(2)}_{aa,0} \\ & + \frac{2n}{(n+2)^2}\left[\delta^{(1)}_{AA,0}\right]^2 \end{aligned}$$

Integrating this equation to solve for the quantity $-2\delta^{(2)}_{AA,1} + n\delta^{(2)}_{Aa,0}$, we obtain

$$\begin{aligned} -2\delta^{(2)}_{AA,1} + n\delta^{(2)}_{Aa,0} = {} & \frac{2n(p - p'r'r^{-1})}{(n+2)^2(2+p)}\left[\delta^{(1)}_{AA,0}\right]^2 \\ & + \frac{2n((2-p)(p - p'r'r^{-1}) + pp'r'r^{n}t)}{(n+2)^2p^2}\left[\delta^{(1)}_{AA,0}\right]^2 \exp(-r^{n+1}t) \\ & - \frac{8n(p - p'r'r^{-1})}{(n+2)^2p^2(2+p)}\left[\delta^{(1)}_{AA,0}\right]^2 \exp\left(\frac{-(2+p)}{2}r^{n+1}t\right) \end{aligned} \tag{43}$$

The queen-control allele invades a resident wild-type population if

$$\lim_{\substack{\epsilon t \to 0 \\ t \to \infty}} \dot{\delta}^{(2)}_{AA,0} > 0 \tag{44}$$

Substituting (39), (40), (41), (42), and (43) into (44), we find that the recessive allele for

queen control of worker reproduction increases in frequency if

$$\frac{r'}{r} > \frac{2(1+p)}{2+p+p'} \tag{45}$$

Condition (45) can be written more intuitively:

$$\frac{r'}{r} > 1 - \frac{p'-p}{2+p+p'}$$

A.3 Stability of a Dominant Queen-Control Allele

Suppose that we initially have a population in which all queens suppress their workers' reproduction. If we introduce a small amount of the allele for no queen control, A, and if the queen-control allele, a, is dominant, then is queen control evolutionarily stable to being undone by non-controlling queens?

Based on what we already have, the evolutionary stability condition for a dominant queen-control allele is obtained readily using a simplified procedure. Notice that the calculations of Section A.2 for invasion of a recessive queen-control allele describe the following scenario: We begin with a homogeneous population of colonies, where all individuals are homozygous for the A allele. A fraction p of males originate from the queen, and each colony's reproductive efficiency is r. The mutant allele's only effects are to change the value of p' relative to p and to alter the colony efficiency r' relative to r. Here, p and r are the fraction of queen-derived males and the colony efficiency, respectively, for colonies headed by type AA and type Aa queens, while p' and r' are the corresponding parameters for colonies headed by type aa queens.

Then, consider the evolutionary stability of a dominant a allele for controlling queens. We again begin with a homogeneous population of colonies, but in this case, all individuals

are homozygous for the a allele. A fraction p' of males originate from the queen, and each colony's reproductive efficiency is r'. The mutant allele's only effects are to change the value of p relative to p' and to alter the colony efficiency r relative to r'. Here, p' and r' are the fraction of queen-derived males and the colony efficiency, respectively, for colonies headed by type aa and type Aa queens, while p and r are the corresponding parameters for colonies headed by type AA queens.

Therefore, if we take Condition (45), swap p and p', swap r and r', and reverse the sign of the inequality, then we obtain the condition for evolutionary stability of a dominant queen-control allele:

$$\frac{r'}{r} > \frac{2+p+p'}{2(1+p')} \tag{46}$$

Condition (46) can be written more intuitively:

$$\frac{r'}{r} > 1 - \frac{p'-p}{2(1+p')}$$

A.4 Stability of a Recessive Queen-Control Allele

Suppose that we initially have a population in which all queens suppress their workers' reproduction. If we introduce a small amount of the allele for no queen control, A, and if the queen-control allele, a, is recessive, then is queen control evolutionarily stable to being undone by non-controlling queens?

Based on what we already have, the evolutionary stability condition for a recessive queen-control allele is obtained readily using a simplified procedure. Notice that the calculations of Section A.1 for invasion of a dominant queen-control allele describe the following scenario: We begin with a homogeneous population of colonies, where all individuals are homozygous for the A allele. A fraction p of males originate from the queen, and each colony's reproductive efficiency is r. The mutant allele's only effects are to change the value of p' relative to p and

to alter the colony efficiency r' relative to r. Here, p and r are the fraction of queen-derived males and the colony efficiency, respectively, for colonies headed by type AA queens, while p' and r' are the corresponding parameters for colonies headed by type Aa and type aa queens.

Then, consider the evolutionary stability of a recessive a allele for controlling queens. We again begin with a homogeneous population of colonies, but in this case, all individuals are homozygous for the a allele. A fraction p' of males originate from the queen, and each colony's reproductive efficiency is r'. The mutant allele's only effects are to change the value of p relative to p' and to alter the colony efficiency r relative to r'. Here, p' and r' are the fraction of queen-derived males and the colony efficiency, respectively, for colonies headed by type aa queens, while p and r are the corresponding parameters for colonies headed by type AA and type Aa queens.

Therefore, if we take Condition (31), swap p and p', swap r and r', and reverse the sign of the inequality, then we obtain the condition for evolutionary stability of a recessive queen-control allele:

$$\frac{r'}{r} > \frac{2+p+p'}{2(1+p')} \tag{47}$$

Condition (47) can be written more intuitively:

$$\frac{r'}{r} > 1 - \frac{p'-p}{2(1+p')}$$
